# Relationship between Cognitive Functions and Sport-Specific Physical Performance in Youth Volleyball Players

**DOI:** 10.3390/brainsci11020227

**Published:** 2021-02-12

**Authors:** Athos Trecroci, Marco Duca, Luca Cavaggioni, Alessio Rossi, Raffaele Scurati, Stefano Longo, Giampiero Merati, Giampietro Alberti, Damiano Formenti

**Affiliations:** 1 Department of Biomedical Sciences for Health, Università degli Studi di Milano, 20133 Milano, Italy; Athos.Trecroci@unimi.it (A.T.); marco.duca@unimi.it (M.D.); Luca.Cavaggioni@unimi.it (L.C.); Raffaele.scurati@unimi.it (R.S.); stefano.longo@unimi.it (S.L.); giampietro.alberti@unimi.it (G.A.); 2 IRCCS Istituto Auxologico Italiano, Obesity Unit and Laboratory of Nutrition and Obesity Research, Department of Endocrine and Metabolic Diseases, 20149 Milano, Italy; 3 Department of Computer Science, University of Pisa, 56126 Pisa, Italy; alessio.rossi2@gmail.com; 4 Department of Biotechnology and Life Sciences (DBSV), University of Insubria, 21100 Varese, Italy; giampiero.merati@uninsubria.it; 5 IRCCS Fondazione Don Carlo Gnocchi, 20121 Milano, Italy

**Keywords:** young athletes, cognition, team sports, sport-specific skills, motor skills, executive functions

## Abstract

The aim of this study was to investigate the relationship between basic cognitive functions and sport-specific physical performance in young volleyball players. Forty-three female volleyball players (age 11.2 ± 0.8 years) were tested for cognitive performance by measuring simple reaction time (clinical reaction time), executive control (Flanker task), and perceptual speed (visual search task). Moreover, a set of tests was used to assess physical abilities as volleyball-specific skills (accuracy of setting, passing, and serving) and motor skills (change of direction, vertical jump, and balance). A cumulated value for both cognitive and sport-specific physical performance tests was computed by adding up each test’s domain outcomes. Pearson’s r correlation analysis showed a large positive correlation (*r* = 0.45, d-value = 1.01) of the cumulated score summarizing cognitive functions with the cumulated score summarizing sport-specific physical performance. Moreover, small-to-medium correlations (d-value from 0.63 to 0.73) were found between cognitive and motor skills. Given the cumulative scores, these results suggest that volleyball athletes with superior basic cognitive functions present better sport-specific physical performance. Our findings encourage to extend the knowledge of the associations between cognitive and motor skills within a sports performance context.

## 1. Introduction

Volleyball is an intermittent team ball sport requiring players to have well-developed physical and physiological capacities [1] and significant motor control and cognitive functioning. As an open skill sport, sport-specific motor actions are performed in a relatively dynamic and changing environment, which suggests the involvement of high perceptual–cognitive demands during a volleyball match [2]. Recent reviews provided evidence of superior general cognitive functions in expert athletes of team sports over non-expert peers [3,4]. The importance of cognitive functions in open skill sports has been demonstrated especially in soccer, in which high-level players demonstrated better cognitive abilities than their low-level counterparts [5,6]. In addition, better cognitive abilities were found to be associated with future performance success in young soccer players [7,8]. Similar results were also found for volleyball, where elite players demonstrated better cognitive functions than non-athlete controls [9]. A recent study in volleyball has adopted a multidimensional approach to investigate potential differences between players of different competitive levels [10]. Volleyball-specific skills, change of direction (COD ability), vertical jump, and general cognitive functions (executive control and perceptual speed) were found to be superior in players competing at a higher level compared to their low-level peers [10]. Taken together, these findings suggest that general cognitive functions are important components of performance in sports, especially in team sports, where perceptual–cognitive demands are high [9]. However, a combination of both physical and motor performance with basic cognitive functions should be considered to depict a complete portrait of athletes’ abilities [10,11]. In the same context, a further study investigated the relationship between cognitive functions and soccer-specific motor skills in soccer players ranging from 11 to 13 years of age [11]. The attention window was positively correlated with dribbling skills, and working memory was positively associated with dribbling, ball control, and ball juggling skills. Interestingly, the cumulated score of the cognitive tests was positively related to the cumulated score of the motor tests [11]. This finding supports the close interplay of motor and cognitive skills, suggesting a connection between physical and cognitive domains in youth athletic development. In a broader view, apart from a field-based performance context, the health and growth of youngsters (children and adolescents) engaging in physical activity can be harmonically promoted [12,13] by targeting the whole area of well-being including social interactions, active lifestyle, cognition, physical fitness, and motor skills. In a specific view, motor skills and general cognitive functions can share common bases concurring to enhance a holistic development both in athlete and in non-athlete youngsters.

In contrast to the relationship between general cognitive functions and sport-specific motor skills in young soccer players [11], to the best of our knowledge, no studies have examined the association between general cognitive functions and sport-specific motor skills in youth volleyball. Similar to Scharfen and Memmert (2019), the present study intends to extend knowledge on the relationship between general cognitive functions and motor skills in young athletes. This would promote research and discussion on the potential interplay between cognition and motor skills as determinants of sport (especially open skill sports) performance in youth development.

Therefore, the aim of the present study was to investigate the association of basic cognitive functions (simple reaction time, executive control, and perceptual speed) with sport-specific physical performance, including volleyball-specific skills (accuracy of setting, passing, and serving) and motor skills (COD, vertical jump, and balance) in youth volleyball players. These variables were chosen as a previous investigation showed their importance in discriminating volleyball players of different competitive levels [10].

## 2. Materials and Methods

### 2.1. Participants

Fifty-seven young female volleyball players were recruited from the youth academy of three Italian volleyball teams. Players had been practicing volleyball for at least two years. This study was conducted during the second half of the competitive season, from April to May. Experimental procedures were performed in an indoor facility at the same time of the day (i.e., from 3:00 to 6:00 p.m.) under controlled environmental conditions. Fourteen datasets were excluded due to missing cognitive dataset, motor dataset, or both, thus leading to a final sample of 43 participants (age = 11.2 ± 0.8 years, height = 1.52 ± 0.10 m, body mass = 40.5 ± 7.5 kg). Before the commencement of data collection, players’ legal guardians provided written consent after being deeply informed about potential risks and benefits of the study. The study was carried out in accordance with the Helsinki Declaration of 1975 and was approved by the ethics committee of the Università degli Studi di Milano (approval number 2/12).

### 2.2. Procedures

Two testing sessions were scheduled within one week, separated by at least 48 h. The first testing session aimed to assess anthropometric characteristics and cognitive performance, including simple reaction time, executive control, and perceptual speed. Regarding the anthropometric measures, body mass and sitting height were also obtained to compute the corresponding maturity index as from Mirwald and colleagues [14] (Table 1). The second testing session aimed to assess volleyball-specific skills and motor skills. A recovery period of 10 min was allowed between each test to avoid fatigue-induced effects [15]. Participants were familiarized with the testing procedures.

### 2.3. Cognitive Functions

Cognitive functions included a test to assess simple reaction time in a clinical setting and two computer-based tasks to assess executive control and perceptual speed.

- Simple reaction time. Participants’ reaction time was assessed using the clinical reaction time test [16]. During the testing procedure, participants sat at a table, with the dominant limb’s hand open and placed at the table’s edge. The apparatus to measure the reaction time was suspended vertically, the weighted disk aligned with the top of the participant’s open hand. Participants were asked to maintain their gaze on the weighted disc. At random intervals, the examiner released the apparatus, and the participant had to catch the apparatus as quickly as possible. The distance from the top of the disk to the most prominent part of the participant’s hand was measured and converted to clinical reaction time. Participants completed eight trials; the mean value was calculated and served for subsequent analysis.
- Executive control. For this test, a modified version of the Flanker task with arrows was used [17]. Participants were requested to respond as quickly and accurately as possible to the direction of a left or right target arrow while ignoring two flanking arrows on each side, pointing towards the same or the opposite direction. The task included trials in two different conditions, i.e., congruent and incongruent. In the congruent condition, the target and flanking arrows pointed to the same direction (left: < < < < < or right: > > > > >), while in the incongruent condition, the flanking arrows pointed to the opposite direction of the target ones (left: < < > < < or right: > > < > >). When the arrows appeared on the computer screen, participants had to detect the target arrows’ direction and press as quickly as possible the keys A or L for left and right directions, respectively. For each condition, 50 trials were presented randomly with right and left target arrows occurring with the same probability, for a total of 100 trials. Participants had to respond to the target arrow within 2 s after they appeared on the screen. Mean response time was computed for each condition, considering only correct responses. Flanker interference was calculated by subtracting congruent from incongruent trials’ response times and was considered the outcome variable.
- Perceptual speed. The visual search task [18]. was used to measure the perceptual speed. Different stimuli were presented on a computer screen, i.e., a target (an orange letter T) and distractors (blue and/or upside-down orange Ts). Participants had to press the keyboard’s space button when the target stimulus appeared among distractor stimuli. They had to avoid pressing the key if the target was missing. The target stimulus could be present among the 5, 10, 15, and 20 items composing each trial, randomized across a total of 100 trials. For each item trial, 25 trials were presented randomly, with the present or absent target occurring with the same probability. Participants had to respond as quickly and accurately as possible within 4 s from the trial presentation. Mean response time was computed for each item trial considering only correct responses. The sum of the response times for the 5, 10, 15 and 20 items was considered as the outcome variable (visual search total time).

### 2.4. Sport-Specific Physical Performance

#### 2.4.1. Volleyball-Specific Skills

Participants were tested on the accuracy of setting, serving, and passing skills in an indoor facility [19,20], following the procedures adopted in a previous study [21]. Two digital cameras (240 fps, 1080 p, FDR1000V, Sony, Tokyo, Japan) were used to film each skill. Skills’ accuracy was based on the participants’ ability to hit specific targets. Six trials for each skill were performed. For each skill, the sum of points of the six trials was considered as the overall score.

- Setting. The player was positioned close to the net and received the ball from the coach located at a distance of 5 m. The player had to set the ball to a circular target (diameter = 0.8 m) placed in a square (side length = 2.3 m). Setting the ball in the circular target awarded 3 points, hitting the edge of the circle 2 points, placing the ball outside of the circle and inside the square 1 point, and placing it outside of the square 0 points.
- Serving. The player had to serve in the opposite half-court (width = 9 m) from a service position. One point was awarded when the ball reached the opposite half-court, 0 points when a serve fell outside the opposite half-court.
- Passing. The player had to direct a pass from the coach to a target (length = 1.6 m; width = 2.3 m) positioned at the net 2 m from the right-hand sideline. This target was chosen because it was the approximate position of the setter during a match. The coach, positioned in the opposite field 1 m above the ground and 6 m from the net, threw an overhead pass to the receiving player. The player was required to pass the ball to another player standing with the arms extended above their head (setter) in the primary target area. A secondary target area was created, extending from the right-hand sideline (length = 3 m; width = 4.1 m). A pass in the primary target area awarded 2 points, in the secondary target area 1 point, and outside the target area 0 points. For a complete description of the testing battery, please refer to the literature [19,20]. The sum of the accuracy score of the three skills was considered as the outcome variable.

#### 2.4.2. Motor Skills

Motor skills tests included the assessment of COD, vertical jump, and balance.

- COD. The modified agility T-test was used to assess COD speed [22]. Participants began with both feet behind the starting line. At participants’ discretion, they sprinted towards a cone set 5 m in front of them and touched it with the right hand. Then, participants performed a lateral shuffle to the left towards a cone set at a distance of 2.5 m and touched it with the left hand. Then, they shuffled towards a cone set 5 m to their right and touched it with the right hand. After that, they shuffled to their left for 2.5 m and touched the first cone again with the left hand. Finally, they ran backwards and crossed the starting line. Each participant completed two trials, separated by a recovery of 3 min. Participants were required to face forward continuously and not to cross their feet; the inability to comply with these instructions determined the trial’s invalidation. In this case, an additional trial was completed. The average time of two successful trials was included in the analysis. Performance time was recorded using a timing gate system (Witty, Microgate, Bolzano, Italy) [15,23].
- Vertical jump. Participants performed three countermovement jumps (CMJ) with arm swing. An infrared-validated device (Optojump next system, Microgate, Bolzano, Italy) was used to measure vertical jump height [24]. Each participant completed three jumps, observing a recovery period of 3 min between each trial. The average jump height was used for the subsequent analysis [23].
- Balance. Participants performed the balance error scoring system (BESS) test [25,26]. They were instructed to maintain three stances (bilateral, unilateral, and tandem stances) on both a firm and a foam pad. In the bilateral stance, both feet were in contact with the floor, with the internal malleoli closed to each other. In the unilateral stance, the body was supported by the non-dominant limb, while the dominant one was kept off the ground with the hip flexed at 20° and the knee flexed at 45° [23]. In the tandem stance, the foot of the dominant limb was positioned in front of the non-dominant one, with the latter touching the heel of the dominant foot with the toe. Each participant was instructed to keep a stable position for 20 s in each stance. Participants were filmed to allow a posteriori evaluation for reliability and performance purposes. The number of errors detected during each stance was recorded according to previous protocols [23,26]. In case of multiple errors at once, a single error was counted. The BESS test score was obtained by summing the total errors recorded on the firm and foam pad surfaces.

### 2.5. Data Analysis

A cumulated value for all cognitive tests was computed by adding up the outcomes of each cognitive test (cognitive score). Similarly, a cumulated value for the sport-specific physical performance tests was computed by adding up the outcomes of each volleyball-specific and motor skills (motor score). All values were z-standardized before this calculation. This approach was previously adopted in a similar study in soccer players [11]. For temporal outcome variables (COD, simple reaction time, Flanker interference, visual search total), the inverse of the z-scores was computed so that positive values corresponded to better performances in accordance with the z-scores for the remaining variables.

### 2.6. Statistical Analysis

Normality of data distribution was tested using the Shapiro–Wilk’s test. Relative and absolute intra-session reliability of cognitive functions, volleyball-specific skills, and motor skills was assessed using the intra-class correlation coefficient (ICC) [27] and the coefficient of variation (CV), respectively. Intra-rater reliability for the BESS score was assessed using both ICC and CV. Pearson’s r correlation coefficient was used to investigate the correlation between the players’ cognitive, volleyball-specific, and motor skills test results. Moreover, effect sizes (ES) were computed for each correlation coefficient using Cohen’s *d* transformation of r into a d-value according to Equation (1) [28]:

(1) $$d=\frac{2\cdot r}{\sqrt{1-r^{2}}}$$

ES were interpreted as null (<0.2), small (0.2–0.5), medium (0.5–0.8), and large (>0.8). The level of significance was set at *p* ≤ 0.05. Statistical analysis was performed using SPSS v21.0 (IBM, Chicago, IL, USA) and a customized Excel worksheet (Microsoft, Redmond, WA, USA).

## 3. Results

Excellent relative and absolute reliability was found for visual search total time (ICC = 0.84 [0.76–0.9]; CV = 15%), flanker task congruent (ICC = 0.93 [0.89–0.95]; CV = 16%), and flanker task incongruent (ICC = 0.91 [0.87–0.94]; CV = 16%). Also, the clinical reaction time showed a good reliability (ICC = 0.8, 95% CI [0.72–0.87]; CV = 13%). Moreover, good-to-excellent relative and absolute reliability was obtained for volleyball-specific skills (ICC = 0.84, 95% CI [0.78–0.89]; CV = 20%), COD (ICC = 0.91, 95% CI [0.84–0.95]; CV = 13%), CMJ (ICC = 0.94, 95% CI [0.91–0.96]; CV = 5%), BESS (ICC = 0.91, 95% CI [0.87–0.94]; CV = 3%). The descriptive statistics of each outcome variable are shown in Table 1.

**Figure 1:**
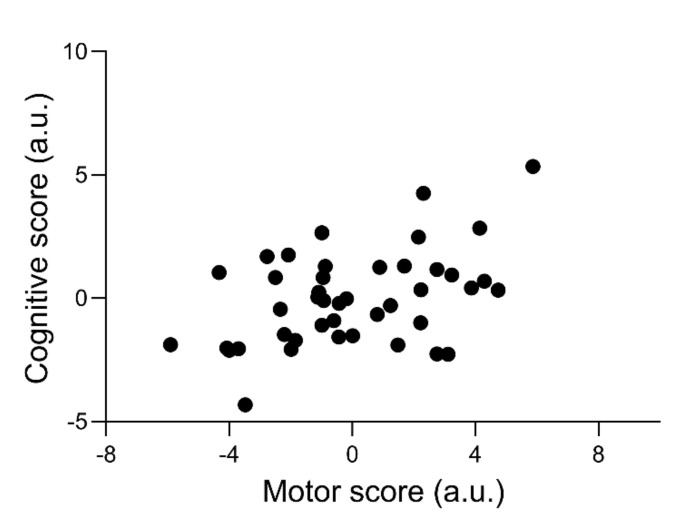
Scatterplot showing the relationship between cognitive score and motor score (*r* = 0.451, *p* = 0.002).

Firstly, a significant correlation was found between cognitive and motor score (large ES) (Table 2). The cognitive score significantly correlated with the modified agility T-test (medium ES) and CMJ (large ES). The motor score significantly correlated with clinical reaction time (medium ES) and visual search total time (medium ES). Furthermore, significant correlations were also found between CMJ and clinical reaction time (medium ES), CMJ and visual search total time (medium ES), BESS and visual search total time (medium ES). Correlation coefficients, inferential statistics, and ESs are shown in Table 2. Figure 1 shows the relationship between cognitive and motor scores.

## 4. Discussion

The aim of the present study was to investigate the association between basic cognitive functions and sport-specific physical performance in young volleyball players. The main finding was that the cumulated score summarizing cognitive functions showed a large positive correlation with the cumulated score summarizing sport-specific physical performance. Overall, this suggests that volleyball athletes with superior basic cognitive functions (expressed by cumulated cognitive score) presented better sport-specific physical performance (expressed by the cumulated motor score).

This finding is in line with a recent study investigating the relationship between cognitive functions and sport-specific motor skills in young soccer players [11]. In that study, the cumulated score of cognitive tests (measuring attention window, perceptual load, multiple object tracking, and working memory) was found to be associated with the cumulated score of motor tests [11]. Specifically, attention window and working memory were positively correlated with dribbling, ball control, and ball juggling [11]. These results could imply that well-developed cognitive functions may contribute to enhance players’ skills within a game-based context characterized by unpredictable scenarios.

In volleyball, players play in complex and dynamic environments, where they have to pay attention to the ball, teammates, and opponents’ movements. This dynamic environment exacerbates their cognitive demands, prompting the players to cope with the increasing complexity of the game [2,29]. Of note, albeit small-to-moderate d-values, the relationships between volleyball-specific skills and cognitive functions were almost significant (Table 2). This is in contrast to a previous study in which cognitive functions significantly correlated with soccer-specific skills (such as dribbling, ball control, ball juggling) [11]. An explanation for this discrepancy can be found within the features of the sport-specific skills. In the current volleyball-specific skills test, athletes were required to hit specific targets under a spatial constraint, but no temporal constraints were present. However, in the previous study [11], players had to perform dribbling, ball control, and ball juggling tests under both spatial and temporal constraints. The combination of spatial and temporal constraints is a typical feature of uncertainty conditions such as those found in open skill sports, where perceptual–cognitive demands are paramount [30]. To assess volleyball-specific skills, the inclusion of temporal constraints within a test (together with spatial constraints) would better reflect the perceptual–cognitive demands of a game compared to a test including only spatial constraints, as done in this study [19,20]. It is conceivable to assume that this might contribute to further the association between volleyball-specific skills and cognitive functions. Nevertheless, to the best of the authors’ knowledge, the present testing battery (including only a spatial constraint) is the only valid and suitable system to assess volleyball-specific skills in studies investigating the exercise–cognition relationship [10,21].

The importance of general cognitive functions in volleyball has been widely demonstrated in previous studies [9,10,21,31]. The combination of cognitive functions (executive control and perceptual speed) and volleyball-specific skills was found to be useful for discriminating players of different competitive levels [10]. Of note, the current cumulated cognitive score was based on tasks assessing executive control and perceptual speed [10], as well as on clinical reaction time [21]. Parallelly, the current cumulated motor score was composed of scores obtained from tests assessing key performance components. Specifically, COD performance (assessed by the agility T-test), vertical jump (assessed by CMJ), and volleyball-specific skills involve key aspects for volleyball players [10,20,32]. Also balance (assessed by the BESS test) is an important component of physical performance because it is required to safely accomplish the execution of any specific movement pattern, especially during the landing phase after a vertical jump [33,34]. Nevertheless, besides the cumulated scores, only a few significant medium relationships were found between each motor and cognitive test (i.e., of CMJ with visual search total time and clinical reaction time, BESS test with visual search total, Table 2). Indeed, due to the multidimensionality and complexity of volleyball, considering only a single component of physical performance may be less informative on the relationship with the cognitive dimension.

The present study assessed motor skills relevant for sports performance in youth athletic development (i.e., volleyball) as done in the study by Scharfen and Memmert on soccer [11]. The finding of a positive association between cognitive and sport-specific performance domains is in line with the large body of literature reporting a substantial relationship between general motor and cognitive skills in children [35], adolescents [36], and adults [37]. Indeed, besides the wellestablished relationship between general measures of cognitive and motor functions, our findings provide evidence of a close interplay between cognitive and motor skills in a sports performance context, suggesting a connection between physical and cognitive domains in youth athletic development [11].

A further insight from the present study regards the mutual relationship between cognitive and motor neural networks [38,39]. Although speculative, the association found in the current study may be partly explained by shared underlying neural processes involved in motor and cognitive tasks. In this regard, Diamond proposed the notion of synchronous activation of the cerebellum and prefrontal cortex in both motor and cognitive tasks to explain the relationship between the motor and the cognitive domains [40]. Consequently, these structural and functional links between neural networks underpinning cognitive and motor tasks might contribute to explain—at least partially—the positive association between the two cumulated scores.

Besides supporting the theoretical association between the cognitive and the motor domains, our findings could also be useful from a practical perspective. For example, in the light of the evidence previously derived from the cognitive component skills approach [3,4,9], focusing on the importance of cognitive functions for sport performance may help coaches scout for new talented volleyball players in a more multidimensional way. Adding cognitive tests to assess players’ physical and motor performance may provide coaches with a more comprehensive picture of the players’ profiles in terms of talent selection. Moreover, this could also help coaches and technical staff to monitor the psycho-cognitive state of their athletes throughout a season including training sessions, official competitions, and recovery sessions.

This study presents some limitations that should be acknowledged. First, as a correlational study aiming to assess the associations between general cognitive functions and sport-specific physical performance in volleyball, a causal relationship between cognitive and motor domains should be investigated using longitudinal study designs. Second, the study employed a relatively small sample size. Additionally, we tested only female athletes, and results cannot be surely extended also to male subjects. Cognitive functions are influenced by many variables, such as age [41] and sex [42]. For these reasons, any form of generalization should be avoided, and results interpreted with caution. Finally, to assess volleyball-specific skills, we adopted tests with spatial constraints only. The combination of spatial and temporal constraints would mirror sport-specific perceptual–cognitive demands. However, given its cross-sectional nature, the present study contributes to providing preliminary knowledge on the interplay between physical and cognitive performance for youth athletic development, laying the foundations for further investigations on the topic. In this respect, an improved understanding of such an interplay could also be helpful for promoting a holistic development approach to health and growth [12,13].

## 5. Conclusions

In conclusion, we found that combinations of cognitive functions (executive control, perceptual speed, clinical reaction time) and sport-specific physical performances (including COD, vertical jump, balance, volleyball-specific skills) were closely related in youth volleyball athletes. Although this is only a first attempt to understand the relationship between cognitive and motor behaviors (especially in an open skill sport), these findings highlight the importance of expanding the knowledge on the associations between cognitive and motor skills within a sports performance context.

## Figures and Tables

**Table 1:** Descriptive statistics for anthropometric, motor, and cognitive variables.

N = 43	Mean	SD
Age (years)	11.21	0.8
Maturity Offset (years)	-2.5	0.7
Body mass (kg)	40.5	7.5
Stature (m)	1.52	0.1
Agility T-test (s)	7.59	0.38
CMJ (m)	0.27	0.03
BESS test (a.u.)	15.0	5.9
Volleyball-specific skills	21.6	4.4
Clinical reaction time (ms)	252	32
Visual search total time (ms)	4307	625
Flanker interference (ms)	133	69

Note: Sample size (N) = 43; CMJ: countermovement vertical jump with arm swing; BESS: balance error scoring system test; volleyball-specific skills: sum of setting, serving, and passing volleyball skills.

**Table 2:** Correlations between sport-specific physical performance (volleyball-specific skills, COD, vertical jump, balance) and cognitive skills.

	N = 43	Agility T-Test	CMJ	BESS Test	Volley-Specific Skills	Motor Score
**Clinical reaction time**						
	Pearson Correlation	0.247	−0.317 *	0.146	−0.277	−0.354 *
	*p*-value	0.111	0.038	0.349	0.072	0.02
	d-value	0.509	−0.668	0.296	−0.577	−0.757
**Flanker Interference**						
	Pearson Correlation	0.246	−0.121	−0.103	−0.207	−0.169
	*p*-value	0.112	0.441	0.51	0.182	0.279
	d-value	0.51	−0.24	−0.21	−0.42	−0.34
**Visual search total time**						
	Pearson Correlation	0.201	−0.303 *	0.346 *	−0.103	−0.343 *
	*p*-value	0.196	0.049	0.023	0.511	0.025
	d-value	0.41	−0.636	0.738	−0.207	−0.729
**Cognitive score**						
	Pearson Correlation	−0.358 *	0.395 **	−0.209	0.295	0.451 **
	*p*-value	0.019	0.009	0.178	0.055	0.002
	d-value	−0.767	0.860	−0.428	0.617	1.011

Note: sample size (N) = 43; * = correlation was considered significant at the 0.05 level (two-tailed); ** = correlation is significant at the 0.01 level (two-tailed). For temporal outcome variables (change of direction ability (COD), clinical reaction time, Flanker interference, visual search total time), the inverse of the z-scores were computed so that positive values corresponded to better performances in accordance with the z-scores for the remaining variables.

## Data Availability

The data presented in this study are available on request from the corresponding author.
